# Timing of hypoxia PET/CT imaging after 18F-fluoromisonidazole injection in non-small cell lung cancer patients

**DOI:** 10.1038/s41598-022-26199-7

**Published:** 2022-12-16

**Authors:** Pauline Bourigault, Michael Skwarski, Ruth E. Macpherson, Geoff S. Higgins, Daniel R. McGowan

**Affiliations:** 1 Department of Oncology, University of Oxford, Oxford, OX3 7DQ UK; 2 Department of Oncology, Oxford University Hospitals NHS Foundation Trust, Oxford, UK; 3 Department of Clinical Oncology, Guy’s and St Thomas’ NHS Foundation Trust, London, UK; 4 Department of Radiology, Oxford University Hospitals NHS Foundation Trust, Oxford, UK; 5 Department of Medical Physics and Clinical Engineering, Oxford University Hospitals NHS Foundation Trust, Oxford, UK

**Keywords:** Cancer imaging, Lung cancer, Radionuclide imaging, Cancer imaging, Lung cancer

## Abstract

Positron emission tomography (PET)/computed tomography (CT) using the radiotracer 18F-Fluoromisonidazole (FMISO) has been widely employed to image tumour hypoxia and is of interest to help develop novel hypoxia modifiers and guide radiation treatment planning. Yet, the optimal post-injection (p.i.) timing of hypoxic imaging remains questionable. Therefore, we investigated the correlation between hypoxia-related quantitative values in FMISO-PET acquired at 2 and 4 h p.i. in patients with non-small cell lung cancer (NSCLC). Patients with resectable NSCLC participated in the ATOM clinical trial (NCT02628080) which investigated the hypoxia modifying effects of atovaquone. Two-hour and four-hour FMISO PET/CT images acquired at baseline and pre-surgery visits (n = 58) were compared. Cohort 1 (n = 14) received atovaquone treatment, while cohort 2 (n = 15) did not. Spearman’s rank correlation coefficients (ρ) assessed the relationship between hypoxia-related metrics, including standardised uptake value (SUV), tumour-to-blood ratio (TBR), and tumour hypoxic volume (HV) defined by voxels with TBR ≥ 1.4. As the primary imaging-related trial endpoint used to evaluate the action of atovaquone on tumour hypoxia in patients with NSCLC was change in tumour HV from baseline, this was also assessed in patients (n = 20) with sufficient baseline 2- and 4-h scan HV to reliably measure change (predefined as ≥ 1.5 mL). Tumours were divided into four subregions or distance categories: edge, outer, inner, and centre, using MATLAB. In tumours overall, strong correlation (P < 0.001) was observed for SUV_max_ ρ = 0.87, SUV_mean_ ρ = 0.91, TBR_max_ ρ = 0.83 and TBR_mean_ ρ = 0.81 between 2- and 4-h scans. Tumour HV was moderately correlated (P < 0.001) with ρ = 0.69 between 2- and 4-h scans. Yet, in tumour subregions, the correlation of HV decreased from the centre ρ = 0.71 to the edge ρ = 0.45 (P < 0.001). SUV, TBR, and HV values were consistently higher on 4-h scans than on 2-h scans, indicating better tracer-to-background contrast. For instance, for TBR_max_, the mean, median, and interquartile range were 1.9, 1.7, and 1.6–2.0 2-h p.i., and 2.6, 2.4, and 2.0–3.0 4-h p.i., respectively. Our results support that FMISO-PET scans should be performed at 4 h p.i. to evaluate tumour hypoxia in NSCLC.

**Trial registration:** ClinicalTrials.gov, NCT02628080. Registered 11/12/2015, https://clinicaltrials.gov/ct2/show/NCT02628080.

## Introduction

The prognosis of patients with non-small cell lung cancer (NSCLC) is poor despite advances in the delivery of several treatment modalities. Solid tumours like NSCLC generally rely on a dysfunctional vasculature for oxygen delivery^1^. Associated with their high metabolic demand, this causes tumour hypoxia. It is well established that tumour hypoxia induces resistance to numerous anticancer treatments, and this is particularly pertinent for radiotherapy (RT)^2^. There is therefore much interest in imaging tumour hypoxia to develop novel hypoxia modifiers as radiosensitisers, as well as to guide radiation treatment planning.

Chapman et al. detected for the first time in 1981 tumour hypoxia with molecular imaging and nitroimidazole compounds^3^. These exogenous and hypoxia-specific markers are reduced and re-oxidised in normoxic cells but, in hypoxic cells, their nitro radical anion is further reduced, and the compounds covalently (and irreversibly) bind to intracellular macromolecules. As pO_2_ values decrease, the degree of reduction augments and pO_2_ levels less than 10 mgHg initiate this reduction^4^.

PET radiotracers include nitroimidazole compounds such as EF5^5^, FAZA^6^, FMISO^7,8^, HX4^9^, FETNIM^10^, and non-nitroimidazole compounds such as ATSM^11^. Since its development in 1989^12^, numerous pre-clinical and clinical studies^7,8,13,14^ have identified FMISO as the most promising method for hypoxia quantification and to date, it’s the most widely studied hypoxia tracer^15,16^. Valk et al.^7^ and Koh et al.^8^ first indicated that FMISO enables hypoxia detection in human tumours. Rasey et al.^14^ also demonstrated the sensitivity of FMISO as a hypoxic marker, and validated the variability, presence and prevalence of tumour hypoxia in 37 patients. Importantly, Gagel et al.^17^ reported a correlation between FMISO uptake and Eppendorf pO_2_ probe measurements which suggests that the tracer is representative of intracellular pO_2_, and this was not observed with FDG^18^. Statistically significant correlations were moreover shown between FMISO uptake and HIF-1α^19^ as well as Pimonidazole immuno-histochemistry staining^20^. Evidence also showed that FMISO can detect hypoxia in the clinical setting in various tumour types including head and neck (H&N) cancer^14^, NSCLC^21^, breast cancer^22^, glioma^23^, and soft-tissue sarcoma^24^.

FMISO is a lipophilic molecule and diffuses passively across cell membranes. As a nitroimidazole compound, FMISO is reversibly reduced, re-oxidised, and diffuses outside the cell under normoxic conditions. In contrast, under hypoxic conditions, FMISO is further reduced which leads to its irreversible and covalent binding to intracellular macromolecules. FMISO binding occurs at rates inversely proportional to pO_2_ levels in cells, and its uptake increases markedly as the oxygen concentration drops to values at which the oxygen enhancement ratio (OER), and thus tumour radiation response, also declines^25^.

Passive diffusion causes FMISO to slowly clear from normoxic cells and slowly accumulate in hypoxic cells. Imaging hypoxia at late time-points is therefore required given the low tracer-to-background contrast^26^. Kobayashi et al. showed that 4-h post-injection (p.i.) was preferred to 2-h p.i. for patients with a brain tumour^27^. Studies performing static imaging of lung cancer with FMISO-PET have acquired scans after at least 2 h p.i.^28,29^. Moreover, several quantitative values in FMISO-PET at 4-h p.i. were reported as reproducible^30^. Yet, whether these values could be equivalent at 2- and 4-h p.i. is uncertain. In H&N cancer, Abolmaali et al. indicated that scans conducted at 4-h after FMISO injection showed greater contrast than those performed at 2-h p.i.^31^, while recently, Kawamura et al. reported a significant correlation between the quantitative values at 2- and 4-h^32^. Thus, the optimal post-injection timing to image NSCLC tumour hypoxia with FMISO remains to be confirmed.

In this study, we hypothesised that hypoxia-related metrics from FMISO-PET scans performed at 2-h p.i. may correlate with the values from FMISO-PET scans performed at 4-h p.i. in the context of NSCLC. This was to investigate whether similar results could be obtained at 2 h p.i. to improve the patient experience and practical aspects of FMISO scanning. The aim was therefore to assess the relationship between quantitative values in FMISO-PET obtained at 2- and 4-h p.i. in patients with NSCLC.

## Methods

### Patients

Patients with resectable NSCLC were recruited for the open-label, nonrandomized, two-cohort ATOM clinical trial (NCT02628080, registered 11/12/2015) completed in accordance with the provisions of the Declaration of Helsinki and Good Clinical Practice guidelines. Ethical approval was obtained from National Research Ethics Service Committee South Central Oxford B (16/SC/0012). All patients provided written informed consent. Patients in cohort 1 (n = 14) received oral atovaquone (Wellvone, 750 mg/5 mL micronized suspension, GlaxoSmithKline) twice daily. Patients were asked to take atovaquone orally together with fat-containing food to aid absorption. Patients in cohort 2 (n = 15) did not receive atovaquone. Eligible patients were aged ≥ 18 years, had a pathologic or radiological diagnosis of NSCLC, were scheduled for surgical resection, had disease > 2 cm in diameter, and had Eastern Cooperative Oncology Group (ECOG) performance status 0–2. Patients were excluded if taking known electron transport chain inhibitors. Despite a male predominance in untreated patients, the main clinical characteristics were well balanced in the two cohorts. For full details regarding trial design and patient baseline characteristics, the reader is referred to Ref.^33^. The clinical characteristics of the patients are shown in Table 1 of Ref.^34^.

**Table 1:** Summary of the correlation analysis of SUV and TBR values in tumours overall on FMISO-2 h and FMISO-4 h hypoxia PET-CT scans for all patients (baseline and pre-surgery visits included).

	SUV_max_		SUV_mean_		TBR_max_		TBR_mean_	
	2 h	4 h	2 h	4 h	2 h	4 h	2 h	4 h
Mean (median) [IQR]	2.9 (2.7) [2.5–3.2]	3.6 (3.3) [2.8–3.9]	2.0 (2.0) [1.8–2.3]	2.1 (2.0) [1.8–2.2]	1.9 (1.7) [1.6–2.0]	2.6 (2.4) [2.0–3.0]	1.0 (1.0) [0.9–1.1]	1.2 (1.2) [1.1–1.4]
Correlation coefficient	0.87		0.91		0.83		0.81	
P-value	< 0.001		< 0.001		< 0.001		< 0.001	

### Data acquisition

Single bed position image acquisition centred on the tumour was performed with GE Discovery 690 or 710 PET-CT Scanners (GE Healthcare) for 10 min at 2 and 4 h following the administration of 18F-Fluoromisonidazole (FMISO) (manufactured by University of Cambridge) with an activity of 370 MBq. Precursor (1-(2′-Nitro-1′-imidazolyl)-2-*O*-tetrahydropyranyl-3-*O*-toluenesulfonyl-propanediol, NITTP) was used from Advanced Biochemical Compounds (ABX) and the synthesis of FMISO was performed on a FASTlab synthesis module (GE Healthcare) according to the manufacturer’s instructions. The product was then sterilized by filtration through a Millex-GV 0.22 µm sterile filter (Merck Millipore)^35^.

The same scanner was used for the two visits of each patient with baseline scans and pre-surgery scans for atovaquone-treated and untreated patients. CT images provided attenuation correction and localization. All PET images were reconstructed with a Bayesian penalised-likelihood algorithm, Q.Clear (GE Healthcare) using a beta value of 400^36^. As with previous work, respiratory motion correction was not performed for the presented analysis^38^. Patients in cohort 1 had a median length of 13.5 (IQR 10.75–14) days between imaging timepoints, depending on their planned date for surgery. Patients in cohort 2 had a median length of 14 (IQR 7–14) days between imaging timepoints.

### Image analysis

Tumours on 4-h hypoxia PET-CT images were manually outlined on the CT image by an experienced radiologist and copied to the co-registered PET image. Two-hour images were rigidly registered (CT-to-CT) to 4-h images using Hermes Hybrid Viewer (Hermes Medical Solutions AB), followed by manual adjustment (matching to the tumour region) when required. The tumour outlines on the 4-h images were used to delineate tumours on the respective 2-h images. A matrix containing each voxel coordinates (x, y, z) along with the respective radiotracer standardised uptake value (SUV_voxel_) per voxel was first extracted for every outlined tumour volume, as previously described^34^.

The background mean SUV (SUV_mean background_) was obtained by outlining blood (using a 10 mm diameter circular region of interest (ROI) on at least ten sequential axial PET slices) in the central portion of the descending aorta, with the mean value the average of all outlined voxels. To measure the hypoxic volume (HV), each tumour voxel’s SUV was divided by SUV_mean background_ to determine the tumour-to-blood ratio (TBR_voxel_) value per voxel:

1 $${\text{TBR}}_{\text{voxel}} \, = \text{ } \frac{{\text{SUV}}_{\text{voxel}}}{{\text{SUV}}_\text{mean background}}.$$

As described by Koh et al.^8^, and previously published^33,37^, voxels with a TBR equal to or greater than 1.4 were classified as hypoxic.

Different metrics describing hypoxia were calculated on the 2-h and 4-h images of all 29 patients, including maximum TBR (TBR_max_), mean TBR (TBR_mean_), maximum SUV (SUV_max_), mean SUV (SUV_mean_), and HV in tumours overall and in tumour subregions. As the primary imaging-related trial endpoint used to evaluate the action of atovaquone on tumour hypoxia in patients with NSCLC was change in tumour HV from baseline, this was also assessed in patients (n = 20) with sufficient baseline HV to reliably measure change (predefined as HV ≥ 1.5 mL)^33,37^ in 2- and 4-h images.

In-house MATLAB (version R2021a, MathWorks, Natick, MA, USA) code was first used to calculate the distance of every tumour voxel to the nearest edge of the outlined tumour. Voxel dimensions on PET-CT images were 2.7 × 2.7 × 3.3 mm^3^. Voxels were then divided into four subregions or distance categories: *edge* (the outermost shell of voxels), *outer* (voxels’ centre up to 5.5 mm of the tumour outline), *inner* (voxels’ centre between 5.5 and 11 mm of the tumour outline), and *central* (voxels’ centre more than 11 mm inside the tumour outline), in line with a previous PET study about FMISO uptake in advanced NSCLC^38^.

### Statistical analysis

Statistical analyses used IBM SPSS Statistics (version 27). The normality of data was inspected using Shapiro–Wilk test. Mean, median, and interquartile range (IQR) were indicated for all hypoxia metrics assessed. Spearman’s rank correlation coefficients (ρ) were reported for correlation analysis of TBR_max_, TBR_mean_, SUV_max_, SUV_mean_, and tumour HV on 2- and 4-h scans. For each distance category, the chi-square test of homogeneity was employed to assess the significance of pre- to post-atovaquone changes in the proportions of voxels assigned to each region. Percentage change in tumour HV was calculated between trial visits for each patient. A *P* value < 0.05 was considered significant.

### Ethics approval and consent to participate

Ethical approval was obtained from National Research Ethics Service Committee South Central Oxford B (16/SC/0012). Trial conduct adhered to all regulatory requirements and was in full accordance with the provisions of the Declaration of Helsinki and Good Clinical Practice guidelines.

## Results

### Evaluation of SUV and TBR values at 2- and 4-h post-FMISO injection

Changes in tumour hypoxia were analysed using 4-h hypoxia PET-CT, according to current standard of practice. We investigated whether similar TBR results could be obtained at 2 h p.i. to improve the patient experience and practical aspects of FMISO scanning. The relationship of hypoxia-related quantitative values in FMISO-PET obtained at 2- and 4-h post-tracer injection were therefore investigated.

**Figure 1:**
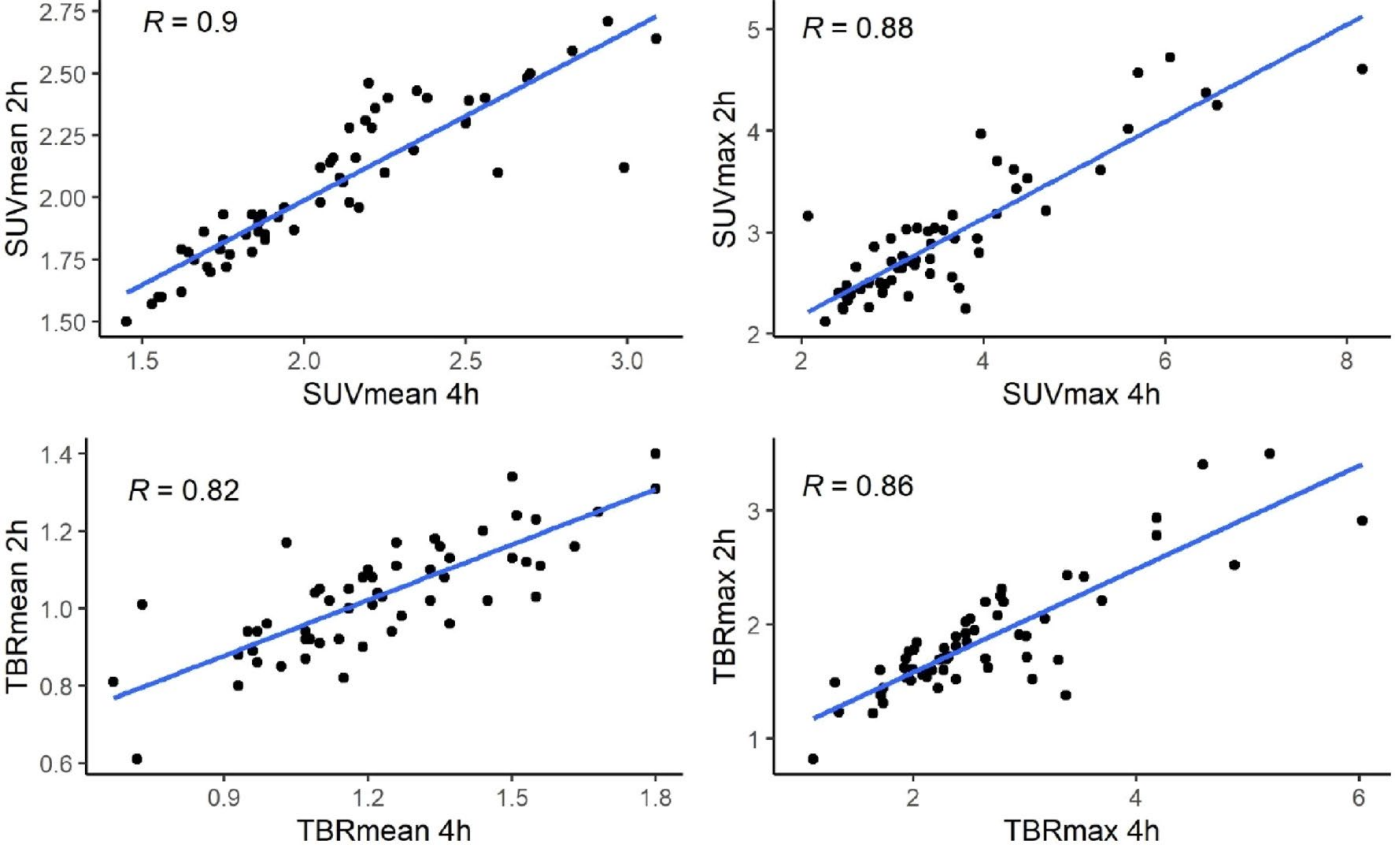
Scatter plots of SUV and TBR values in tumours overall on FMISO-2 h and FMISO-4 h hypoxia PET-CT scans for all patients (baseline and pre-surgery visits included).

The correlations of SUV_max_, SUV_mean_, TBR_max_, TBR_mean_ values in tumours overall and in regions on FMISO hypoxia PET-CT scans at 2- and 4-h were analysed (n = 58 scans compared at 2 and 4 h for all 29 patients, including baseline and pre-surgery visits). Overall, the Spearman’s rank correlation coefficients (ρ) of the parameters were as follows: SUV_max_ (ρ = 0.87), SUV_mean_ (ρ = 0.91), TBR_max_ (ρ = 0.83), and TBR_mean_ (ρ = 0.81). Although, means of these quantitative values were lower on 2-h scans than on 4-h scans, values were highly correlated between scans (*P* < 0.001) (Table 1, Fig. 1). The hypoxia-related quantitative values were also highly correlated in tumour regions on 2- and 4-h scans (Supplementary Table 1).

### Evaluation of tumour hypoxic volumes at 2- and 4-h post-FMISO injection

Overall, in all patients, the Spearman’s rank correlation coefficient (ρ) of tumour HV on 2- and 4-h scans was 0.69 (*P* < 0.001). In the different tumour regions, ρ was also relatively lower, decreasing from the centre (0.71) to the edge (0.45) (*P* < 0.001) (Table 2). As for SUV and TBR values, means of HVs on 4-h scans were higher than on 2-h scans.

**Table 2:** Summary of the correlation analysis of tumour HV overall and in regions on FMISO-2 h and FMISO-4 h hypoxia PET-CT scans for all patients (baseline and pre-surgery scans included).

	Overall		Centre		Inner		Outer		Edge	
	2 h	4 h	2 h	4 h	2 h	4 h	2 h	4 h	2 h	4 h
Mean (median) [IQR]	13.4 (5.0) [0.9–17.2]	30.3 (12.1) [3.1–34.3]	15.4 (2.5) [0.0–21.2]	26.4 (7.2) [0.0–28.2]	22.8 (12.3) [1.8–34.6]	33.2 (9.5) [2.2–40.5]	23.4 (13.2) [1.4–35.8]	34.8 (10.8) [2.9–34.9]	6.4 (0.0) [0.0–4.8]	8.3 (0.4) [0.0–9.1]
Correlation coefficient	0.69		0.71		0.68		0.60		0.45	
p-value	< 0.001		< 0.001		< 0.001		< 0.001		< 0.001	

Given that change in tumour HV was the main imaging-related trial endpoint used to evaluate the action of atovaquone on tumour hypoxia in patients with NSCLC, the percentage change in HV from baseline was assessed on 2-h and 4-h FMISO PET-CT scans. Only 20 patients who had sufficient (≥ 1.5 mL) HV on both their baseline 2- and 4-h scans to reliably detect change were included.

**Figure 2:**
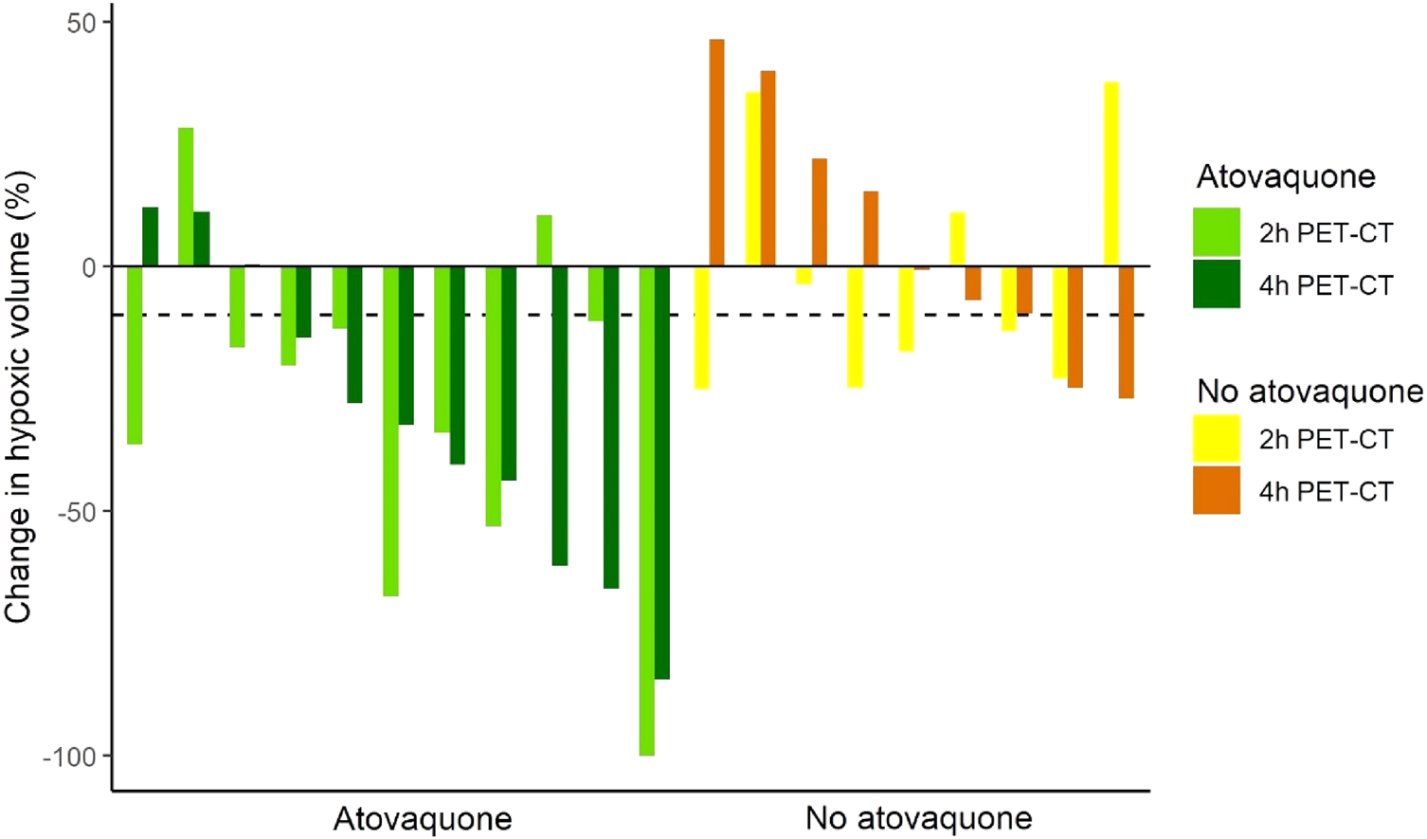
Percentage change in tumour hypoxic volume from baseline on FMISO-2 h and FMISO-4 h hypoxia PET-CT scans for all patients with enough (≥ 1.5 mL) HV on their baseline scans to reliably detect change (n = 20).

A decrease in HV equal to or greater than 10% from baseline was deemed as a meaningful decrease change in HV, as previously described^32,34^. Discrepancies in meaningful changes in HV in three treated patients (27%) and four (44%) untreated patients were observed, as HV increased on 4-h scans and decreased on 2-h scans or vice versa (Fig. 2). Eight (73%) atovaquone-treated patients had an overall and meaningful decrease in HV from baseline according to 4-h scans, whereas nine (82%) treated patients showed a meaningful reduction on 2-h scans. Only two (22%) untreated patients had an overall reduction in HV equal to or greater than 10% on 4-h scans, contrary to five (56%) untreated patients according to 2-h scans. Although SUV and TBR values were highly correlated on 2- and 4-h FMISO PET-CT scans, assessment of the change in tumour HV from baseline highlighted differences between scans.

## Discussion

In order to improve the patient experience and practical aspects of FMISO PET scanning, we investigated whether the hypoxia-related metrics on PET scans could be similar at 2- and 4-h p.i.

A strong correlation was observed for SUV_max_, SUV_mean_, TBR_max_, and TBR_mean_ values between 2- and 4-h scans. This contrasted with the relatively weaker between-scan correlation of HVs, particularly in tumour subregions, and the discrepancies observed in the percentage change in HV from baseline. Notably, HV could form the basis of RT dose escalation or treatment planning in opposition to TBR alone. In this study, the spatial correlation of the hypoxic voxels was not examined, which would matter for RT planning in terms of ‘dose painting’ to deliver higher radiation dose to hypoxic subregions. Mean values for SUV, TBR and HV were considerably higher at 4 h than at 2 h p.i. Indeed, FMISO enters cells through passive diffusion and is then reduced in a two-step process. FMISO binding is reversed in the presence of oxygen, and becomes irreversibly trapped inside the cell in the absence of oxygen. Visualizing hypoxic regions using FMISO consequently takes time because of the tracer’s slow clearance and lipophilicity in normoxic tissues. Given the lower tumour HV and tracer-to-tissue contrast on 2-h scans than on 4-h scans, the assessment of change in HV may be less accurate at 2 h than 4 h p.i. Less tracer-to-tissue contrast also means that a higher cut off to reliably measure change in HV at this timepoint may be needed.

We therefore concluded that performing hypoxia PET scanning at 4 h p.i. seemed more appropriate than at 2 h in patients with NSCLC. Moreover, nine patients (31%), including three treated and six untreated patients, did not have enough baseline HV (i.e. < 1.5 mL) on 2-h scans to reliably measure change in tumour HV from baseline whereas they had sufficient HV on 4-h scans. This would be another argument for scanning at 4 h and not at 2 h p.i., especially when testing novel hypoxia modifiers.

A recent study reported a very strong correlation (n = 20 patients, ρ ≥ 0.96) between all hypoxia-related quantitative values assessed on 2-h and 4-h FMISO PET scans and which supported the use of FMISO PET imaging at 2 h in H&N cancer^32^. In addition to the evaluation of SUV_mean_, SUV_max_, TBR_mean_ and TBR_max_ values between scans, this group compared the metabolic tumour volume and total lesion hypoxia to define tumour hypoxia, instead of the common HV metric that usually includes voxels with TBR ≥ 1.4. Our study showed differences in HV between 2-h and 4-h scans. The authors also suggested that the discrepancy between their study and others, that for instance demonstrated the high reproducibility of SUV_max_, TBR, and HV at 4 h p.i^30^. as well as the higher contrast of FMISO PET scans acquired at 4 h compared to 2 h^31^, could be due to a difference in analysis methodology (absolute values against correlations) and to the greater sensitivity of their PET scanners. Yet, our study, which also used images from a sensitive PET scanner and Spearman’s rank correlations for the analysis, supported the acquisition of PET scans at 4 h for NSCLC due to superior image contrast, in agreement with previously published evidence^30,31^. Changes in hypoxia-related metrics in tumour subregions, not only in tumours overall, were also assessed here.

Moreover, differences in imaging hypoxia using tracers such as FMISO in different tumour types could be due to differences in tumour histology (e.g., squamous cell carcinoma versus adenocarcinoma). Levels of hypoxia may also vary between tumour types as studies reported differences in electrode measurements of pO2 between different types of tumours^39^.

As a shorter waiting time for hypoxia PET imaging would have significant practical and logistical advantages for radiology departments and be more acceptable to patients, the tracers HX4^40,41^ and DiFA^41,42^ have recently been developed and could potentially enable shorter hypoxia PET acquisition times thanks to their greater hydrophilicity. Formal investigation of these tracers is however required in clinical studies.

Our comparison of hypoxia-related quantitative values on FMISO-PET scans at 2 and 4 h included several limitations. First, we assumed that SUV_max_, SUV_mean_, TBR_max_, TBR_mean_ and HV metrics were true on 4-h images and we used them as a reference to compare with 2-h images. Second, patient motion may have impacted the registration of 2-h scans with 4-h ones. Image registration was therefore manually verified for each individual scan. Third, scans were analysed from a small number of patients who received FMISO for hypoxia PET imaging. However, the number of patients is relatively large for this category of imaging study, and represents one of the largest in a single tumour type to date. Collaborative initiatives, such as imaging repositories, may help standardise the methodology used.

## Conclusion

In this study, we compared 4-h and 2-h FMISO-PET scans to assess whether the acquisition time of hypoxia imaging could be reduced in NSCLC patients. Shorter acquisitions would ease the integration of hypoxia PET imaging within hypoxia-targeted therapy trials and potentially adoption into future routine clinical use. A good correlation for hypoxia-related metrics between 2- and 4-h scans was observed at whole tumour and subregion levels. However, there were differences in HV assessments between the two time points at whole tumour and subregion levels. Overall, given the better tracer-to-background contrast at 4 h p.i., our results support that scans should be performed at this timepoint to evaluate tumour hypoxia in NSCLC.

## Supplementary Information

Supplementary Information.

## Data Availability

Data is available under reasonable request to the corresponding author.

## Abbreviations

DiFA: 2,2-Dihydroxymethyl-3-[18F]fluoropropyl-2-nitroimidazole
EF5: [18F]-2-(2-nitro-1[H]-imidazol-1-yl)-*N*-(2,2,3,3,3-pentafluoropropyl)-acetamide
^18^F: Fluorine-18
FAZA: [18F]-fluoroazomycinarabinofuranoside
FDG: [18F]-fluorodeoxyglucose
FMISO: [18F]-fluoromisonidazole
H&N: Head and neck
HV: Hypoxic volume
HX4: [18F]-flortanidazole
IQR: Interquartile range
NSCLC: Non-small cell lung cancer
OER: Oxygen enhancement ratio
PET: Positron emission tomography
p.i.: Post-injection
pO_2_: Partial pressure of oxygen
ROI: Region of interest
RT: Radiotherapy
SUV: Standardised uptake value
SUV_max_: Maximum SUV
SUV_mean_: Mean SUV
SUV_background mean_: Background mean SUV
TBR: Tumour-to-blood ratio
TBR_max_: Maximum TBR
TBR_mean_: Mean TBR
