# Supplementary material for: Timing of hypoxia PET/CT imaging after 18F-fluoromisonidazole injection in non-small cell lung cancer patients
Source: Sci Rep. 2022 Dec 16;12:21746. doi: 10.1038/s41598-022-26199-7 (PMC9758119; doi:10.1038/s41598-022-26199-7)
Supplement: Supplementary file 1 — Supplementary Information. [file 41598_2022_26199_MOESM1_ESM.docx]

**Supplementary Table 1.** Summary of the correlation analysis of SUV and TBR values in tumour centre (**a**), inner (**b**), outer (**c**), and edge (**d**) regions on FMISO-2h and FMISO-4h hypoxia PET-CT scans for all patients (baseline and pre-surgery visits included).

(**a**)

|  | **SUV_max_** | |  | **SUV_mean_** | |  | **TBR_max_** | |  | **TBR_mean_** | |
| --- | --- | --- | --- | --- | --- | --- | --- | --- | --- | --- | --- |
|  | 2h | 4h |  | 2h | 4h |  | 2h | 4h |  | 2h | 4h |
| Mean (median)  [IQR] | 2.2 (2.1)  [1.6-2.5] | 2.5 (2.4)  [1.5-2.9] |  | 1.5 (1.5)  [1.1-1.8] | 1.6 (1.6)  [1.2-1.8] |  | 1.4 (1.3)  [1.0-1.7] | 1.7 (1.6)  [1.1-2.3] |  | 0.9 (0.9)  [0.8-1.0] | 1.1 (1.1)  [0.9-1.3] |
|  |  |  |  |  |  |  |  |  |  |  |  |
| Correlation coefficient | 0.81 | |  | 0.91 | |  | 0.90 | |  | 0.80 | |
| P-value | <0.001 | |  | <0.001 | |  | <0.001 | |  | <0.01 | |

(**b**)

|  | **SUV_max_** | |  | **SUV_mean_** | |  | **TBR_max_** | |  | **TBR_mean_** | |
| --- | --- | --- | --- | --- | --- | --- | --- | --- | --- | --- | --- |
|  | 2h | 4h |  | 2h | 4h |  | 2h | 4h |  | 2h | 4h |
| Mean (median)  [IQR] | 2.5 (2.6)  [2.1-2.9] | 2.9 (2.8)  [2.2-3.3] |  | 1.7 (1.8)  [1.6-1.9] | 1.8 (1.7)  [1.6-2.0] |  | 1.6 (1.5)  [1.3-1.8] | 2.0 (1.9)  [1.5-2.4] |  | 1.2 (1.1)  [1.0-1.2] | 1.3 (1.3)  [1.0-1.5] |
|  |  |  |  |  |  |  |  |  |  |  |  |
| Correlation coefficient | 0.92 | |  | 0.91 | |  | 0.87 | |  | 0.82 | |
| P-value | <0.001 | |  | <0.001 | |  | <0.001 | |  | <0.001 | |

(**c**)

|  | **SUV_max_** | |  | **SUV_mean_** | |  | **TBR_max_** | |  | **TBR_mean_** | |
| --- | --- | --- | --- | --- | --- | --- | --- | --- | --- | --- | --- |
|  | 2h | 4h |  | 2h | 4h |  | 2h | 4h |  | 2h | 4h |
| Mean (median)  [IQR] | 2.6 (2.5)  [2.3-2.9] | 3.1 (2.9)  [2.5-3.3] |  | 1.9 (1.9)  [1.7-2.1] | 2.0 (1.9)  [1.7-2.1] |  | 1.7 (1.6)  [1.5-1.8] | 2.2 (2.0)  [1.8-2.4] |  | 1.3 (1.1)  [0.9-1.2] | 1.3 (1.2)  [1.1-1.5] |
|  |  |  |  |  |  |  |  |  |  |  |  |
| Correlation coefficient | 0.88 | |  | 0.83 | |  | 0.75 | |  | 0.73 | |
| P-value | <0.001 | |  | <0.001 | |  | <0.001 | |  | <0.001 | |

(**d**)

|  | **SUV_max_** | |  | **SUV_mean_** | |  | **TBR_max_** | |  | **TBR_mean_** | |
| --- | --- | --- | --- | --- | --- | --- | --- | --- | --- | --- | --- |
|  | 2h | 4h |  | 2h | 4h |  | 2h | 4h |  | 2h | 4h |
| Mean (median)  [IQR] | 2.4 (2.4)  [2.0-2.7] | 2.7 (2.5)  [2.2-3.4] |  | 0.8 (0.7)  [0.4-1.0] | 0.8 (0.7)  [0.4-0.9] |  | 1.5 (1.5)  [1.1-1.7] | 2.0 (1.9)  [1.4-2.5] |  | 0.5 (0.5)  [0.4-0.6] | 0.6 (0.6)  [0.4-0.7] |
|  |  |  |  |  |  |  |  |  |  |  |  |
| Correlation coefficient | 0.72 | |  | 0.80 | |  | 0.72 | |  | 0.64 | |
| P-value | <0.001 | |  | <0.001 | |  | <0.001 | |  | <0.001 | |
